# Characterising smoking and smoking cessation attempts by risk of alcohol dependence: A representative, cross-sectional study of adults in England between 2014-2021

**DOI:** 10.1016/j.lanepe.2022.100418

**Published:** 2022-06-09

**Authors:** Claire Garnett, Melissa Oldham, Lion Shahab, Harry Tattan-Birch, Sharon Cox

**Affiliations:** a Department of Behavioural Science and Health, University College London, London WC1E 7HB, UK; b Spectrum Consortium, London, UK

**Keywords:** Alcohol dependence, Smoking, Treatment, Tobacco, Cessation, Comorbidities, Classifications, Public health, Health policy

## Abstract

**Background:**

There is a strong shared association between smoking tobacco and drinking alcohol. This study aimed to compare smoking prevalence and smoking characteristics in drinkers who were versus were not at risk of alcohol dependence in England.

**Methods:**

We used cross-sectional data from a monthly, nationally representative survey of adults in England (weighted n=144,583) collected between 2014-2021. Smoking and smoking cessation attempt characteristics were regressed on to alcohol dependence (drinkers at risk versus not at risk), adjusting for survey year.

**Findings:**

Past-year smoking prevalence was 63·3% (95% CI=59·7-66·8) among drinkers at risk of alcohol dependence compared with 18·7% (95% CI=18·4-18·9) among those not at risk, and 19·2% (95% CI=18·8-19·7) among non-drinkers. Among past-year smokers, drinkers at risk of alcohol dependence (versus not at risk) smoked more cigarettes per day (B=3·0, 95% CI=2·3-3·8) and were more likely to smoke their first cigarette within 5 (versus >60) minutes of waking (OR=2·81, 95% CI=2·25-3·51).

**Interpretation:**

In a representative sample of adults in England, a graded effect was observed where smoking prevalence increased with level of alcohol consumption. Past-year smokers at risk of alcohol dependence had higher levels of cigarette dependence than drinkers not at risk. Therefore, smokers at risk of alcohol dependence are a high priority group to target to reduce smoking prevalence as part of the NHS long-term plan.

**Funding:**

Cancer Research UK and the National Institute for Health Research.

**Research in context**

**Evidence before this study**

We searched PubMed up to 16^th^ February 2022 for papers on smoking prevalence among those with alcohol dependence in England, using the terms ("smok*" AND ("alcohol depend" OR "alcohol treat*") AND "England" AND "adults") OR (((cigarette smoking[MeSH Terms]) OR (smoking, cigarette[MeSH Terms])) AND ((abuse, alcohol[MeSH Terms]) OR (alcohol abuse[MeSH Terms])) AND (england[MeSH Terms]) AND (adult[MeSH Terms])). Of the five studies that were identified with these search terms, only one reported smoking prevalence among adults at risk of alcohol dependence in England. This study found that smoking prevalence was 47% among people being treated for alcohol use disorders in 2018/2019, and higher smoking frequency at admission was associated with a relative increase in alcohol use at six months. Whilst substantial reductions in patients’ alcohol use were made during the first six months of treatment, these levels of reduction were not reflected in tobacco use frequency. Other studies identified in the search were not among adults in England and did not report smoking prevalence among those with alcohol dependence.

**Added value of this study**

This study uses a nationally representative survey of adults in the general population in England over an 8-year period to report the smoking prevalence among those at risk of alcohol dependence, and their smoking characteristics. Unlike previous studies, this study reports data from the general population rather than those seeking treatment, providing more generalisable findings of direct relevance to the UK government's aim to reduce smoking prevalence in England to less than 5% by 2030.

**Implications of all the available evidence**

Smoking prevalence is very high among those at risk of alcohol dependence in England, as seen in both the general population (58%) and in those accessing specialist addiction treatment (47%). This indicates the importance of providing targeted smoking cessation support to those at risk of alcohol dependence, including providing smoking and alcohol treatment simultaneously.

Alt-text: Unlabelled box

## Introduction

Smoking is one of the single largest contributors to mortality and morbidity, and contributes to health inequalities.^1^^,^^2^ Smoking is more common and cessation rates are lower among adults facing severe health and social comorbidities,^3^ such as alcohol dependence.^4^^,^^5^ In England, smoking prevalence is an estimated 47% among people seeking treatment for alcohol dependence^4^, though despite this high rate, they are less likely to have their smoking addressed.^4^^,^^6^^,^^7^ The combined use of alcohol and tobacco has a multiplicative effect on the risk of preventable cancers.^8^ Nonetheless, little is known about smoking prevalence, characteristics, and cessation among those people at risk of alcohol dependence in the general population in England, which could inform treatment and practise.

Alcohol dependence exists on a continuum of severity and is defined as a “craving, tolerance, a preoccupation with alcohol and continued drinking in spite of harmful consequences” and associated with an increased rate of mental and physical disorders.^9^ There are over 600,000 people estimated to have alcohol dependence in England,^10^ though only a minority access treatment,^9^^,^^11^ and of these, 40% were receiving treatment for alcohol alongside other substances (e.g. opiate, cannabis).^12^ There is a strong shared association between smoking and drinking^13^ with people who are more dependent on alcohol also being more dependent and heavier smokers.^14^ Alcohol consumption is a known predictor of smoking relapse among people who are heavy drinkers,^15^ and higher levels of alcohol consumption can make quitting smoking more difficult.^16^ However, despite this shared association and a high desire to quit smoking,^6^ those receiving alcohol treatment are less likely to have their smoking addressed.^4^^,^^6^^,^^7^

The lack of smoking cessation support in alcohol dependence treatment has also been attributed to a fear of jeopardising alcohol treatment outcomes, a high prevalence of smoking among treatment staff, and a lack of demonstrated treatment efficacy in this population.^7^ It is also commonly reported that health professionals believe people with competing substance dependencies lack the motivation to quit and that quitting will exacerbate use of other substances.^6^ This not only risks maintaining health inequalities, but it contradicts the advice from several leading health agencies (e.g. Public Health England, US Preventive Services Task Force, and Health Canada) that support should be offered to all smokers, including those from groups with a historically high smoking prevalence rate. There are also a number of advantages of addressing smoking and alcohol dependence simultaneously, such as approaching people when they are open to change (teachable moments), or using relapse prevention skills learned to manage both alcohol and smoking craving.^7^ Furthermore, people who drink alcohol and smoke cigarettes often engage in these behaviours at the same time and laboratory findings suggest that both alcohol and smoking cues can elicit cravings and consumption of the other drug.^17^

However, our understanding of the relationship between those at risk of alcohol dependence and smoking is limited by the clinical context of currently published studies with less known at a population level.^18^^,^^19^ The UK government aims to reduce smoking prevalence in England to less than 5% by 2030. Therefore, it is increasingly important to characterise smoking behaviours among groups who are at increased risk of being excluded or disengaged from mainstream support so that recommendations can be made where to target interventions. This study used data from a nationally representative population survey in England from 2014 to 2021 to describe smoking prevalence rates among adults at risk of alcohol dependence and characterise their smoking and smoking cessation attempt characteristics. We compared smoking and smoking cessation attempt characteristics among past-year smokers who were drinkers at risk of alcohol dependence, drinkers not at risk, and non-drinkers. We also modelled smoking prevalence and smoking and smoking cessation attempt characteristics across the full range of Alcohol Use Disorders Identification Test (AUDIT) scores given that alcohol dependence exists on a continuum of severity.^9^

## Research questions (RQs)

Among adults in England:

1. What is the past-year smoking prevalence: a. drinkers at risk of alcohol dependence, drinkers not at risk, and non-drinkers, and b. across the AUDIT score range for drinkers?
2. What are the smoking and smoking cessation attempt characteristics among those who are past-year smokers who are: a. drinkers at risk of alcohol dependence, drinkers not at risk, and non-drinkers, and b. across the AUDIT score range for drinkers?
3. Do smoking and smoking cessation attempt characteristics of past-year smokers differ between drinkers who are or are not at risk of alcohol dependence?

## Methods

### Study design

The Smoking and Alcohol Toolkit Study is an ongoing, monthly, population survey in England. The Toolkit Study consists of cross-sectional household surveys of nationally representative samples of 1,700-1,800 adults in England.^20^ The study sampling is a hybrid of random probability and simple quota – England is split into more than 170,000 areas stratified according to a geodemographic analysis of the population. Areas are then randomly allocated to interviewers who conduct interviews within that area until the quota is fulfilled.

This study used data from March 2014 (when alcohol questions were first asked) until August 2021 from adults aged 18+. From March 2014 to February 2020, data were collected through face-to-face computer-assisted interviews. No data were collected in March 2020 because of the COVID-19 related social distancing restrictions, and from April 2020 onwards, data were collected via telephone using the same sampling and weighting approach (diagnostic analyses indicate good comparability between collection modalities^21^).

### Sample

In all research questions, the sample consisted of adults aged 18+. In RQs 2 and 3, the sample consisted of those who reported being a past-year smoker, with sensitivity analyses conducted with a sample consisting of those who reported being a current smoker.

People who drink alcohol were defined as having an AUDIT score of 1-40 and non-drinkers as having an AUDIT score of 0.^19^ Those people who drink alcohol were further categorised into those at risk of alcohol dependence as having an AUDIT score ≥20 and those not at risk of alcohol dependence as having an AUDIT score of 1-19. Descriptive statistics for non-drinkers were included in RQs 1 and 2 to provide wider context to smoking prevalence and smoking and smoking cessation attempt characteristics. Non-drinkers were not included in RQ3 where the primary contrast of interest was between drinkers at risk and drinkers not at risk of alcohol dependence, and non-drinkers are a heterogeneous group including both former drinkers (many of whom have given up drinking for health reasons) and long-term or lifetime abstainers.^22^

### Measures

Sociodemographic characteristics measured were: age (continuous); sex (female/male [ref]), and social grade (ABC1 [higher and intermediate professional/managerial and supervisory, clerical, junior managerial/administrative/professional] [ref]/C2DE [skilled, semi-skilled, unskilled manual, and lowest-grade worked or unemployed]).

Current smokers were defined as anyone who reported smoking cigarettes (including hand-rolled), or tobacco of some kind (eg. cigar or shisha) at the time of the survey. Past-year smokers were defined as anyone who was a current smoker (as defined above) or who had stopped smoking completely in the last year. Among past-year and current smokers, the following smoking and smoking cessation attempt characteristics were measured: time to first cigarette (as an indicator of cigarette dependence; more than 60 minutes/30-60 minutes/6-30 minutes/within 5 minutes [ref]^23^); cigarettes smoked per day (continuous); use of roll-your-own tobacco (yes/no [ref]), and any serious quit attempts in the past 12-months (yes/no [ref]). A serious quit attempt was defined to respondents as an attempt to “try to make sure you never smoked again” and to include any current attempt and any successful attempt made within the last year. Of those who had made a serious quit attempt, respondents were also asked: time since start of the most recent quit attempt (last week [ref]/between a week and a month/1-2 months/2-3 months/3-6 months/6-12 months); abrupt versus gradual [ref] quit attempt at most recent attempt; use of evidence-based aids in the most recent quit attempt (e.g. use of e-cigarettes,^24^ nicotine replacement therapies, behavioural support: yes/no [ref]); receipt of GP advice and/or support (yes/no [ref]). Among past-year smokers who had made a serious quit attempt in past 12 months, quit success (having quit smoking completely in the past 12 months) was measured (successful/unsuccessful [ref]). Among current smokers, motivation to quit smoking was measured using the Motivation to Stop Scale,^25^ a continuous score from 1 (‘do not want to stop smoking’) to 7 (‘really want to stop smoking and intend to in the next month’). Full details of the measures included are available in Supplementary File 1.

### Analyses

All analyses were conducted in R with complete cases for all variables of interest. The protocol and analysis plan was pre-registered (https://osf.io/mbqyr/).

### RQ1: past-year smoking prevalence rates

Data were weighted using the rim (marginal) weighting technique to match an English population profile on the dimensions of age, social grade, region, housing tenure, ethnicity, and working status within sex.

Past-year smoking prevalence was reported for drinkers at risk of alcohol dependence, drinkers not at risk and non-drinkers alongside 95% confidence intervals (CIs). Past-year smoking prevalence was modelled as the outcome in a logistic regression model with the full range of AUDIT scores among drinkers as the predictor, adjusted for survey year. To allow for non-linear trends, AUDIT score and survey year were transformed using natural, cubic splines with five and three knots, respectively, placed at equally spaced quantiles of the data. Splines are preferred over categorisation, which neglects that outcomes are more similar in adjacent than distant AUDIT scores or years.^26^ Past-year smoking prevalence was described across the full range of AUDIT scores in a figure and prevalence estimates for AUDIT scores of 5, 10, 15, 20, 25, 30, 35, and 40 reported in a table.

### RQ2: smoking and smoking cessation attempt characteristics among past-year smokers

Unweighted data were used to report the descriptive statistics (mean, SD for continuous variables and %, (n) for categorical variables) of smoking and smoking cessation attempt characteristics for drinkers at risk of alcohol dependence, drinkers not at risk and non-drinkers. Smoking and smoking cessation attempt characteristics were modelled as the outcome in a series of (linear, logistic or multinomial logistic, as appropriate) regression models with the full AUDIT score range among drinkers as the predictor, adjusted for survey year. As for RQ1, natural, cubic splines were applied to allow for non-linear trends.

### RQ3: differences in smoking and smoking cessation attempt characteristics among past-year smokers between drinkers who are or are not at risk of alcohol dependence

Unweighted data were used to compare differences in smoking and smoking cessation attempt characteristics between drinkers who are versus are not at risk of alcohol dependence. Smoking and smoking cessation attempt characteristics were modelled as the outcome in a series of (linear, logistic or multinomial logistic, as appropriate) regression models with risk of alcohol dependence as a predictor, adjusted for survey year (using splines as for RQ1 and 2). For the multinomial logistic regression models, we present the relative risk ratio (RRR) of the coefficient which indicates how the risk of the outcome (categorical variable with more than two values: time to first cigarette and time since start of most recent quit attempt) in the comparison group compared with the reference group changes with the predictor variable (drinkers who are versus are not at risk of alcohol dependence).

### Sensitivity analyses

Sensitivity analyses were conducted for RQ1 where the prevalence of current smoking was reported, and for RQs 2 and 3 where the smoking and smoking cessation attempt characteristics were considered among current, instead of past-year, smokers. For these sensitivity analyses, the motivation to quit variable was included (which is only asked of current smokers).

### Changes from pre-registered analysis plan

The sociodemographic variable ‘housing tenure (owner occupied/other [ref])’ was dropped after pre-registration as the authors became aware that this variable was missing from April 2020 onwards.

Smoking prevalence and smoking characteristics for drinkers not at risk of alcohol dependence were also reported for ease of comparison across the sample and with the non-drinkers and drinkers at risk group.

From October 2020, the Toolkit Study also started to collect monthly data from around 450 and 300 adults in Scotland and Wales, respectively. Past-year and current smoking prevalence among i) drinkers at risk of alcohol dependence, ii) drinkers not at risk, and iii) non-drinkers was also reported in Supplementary File 1 for adults in Great Britain as a whole and stratified by country, for the period that data were available.

### Ethics

Ethical approval for the Smoking and Alcohol Toolkit Studies was granted by the UCL Ethics Committee (ID 2808/005). The data are not collected by UCL and are anonymised when received by UCL.

### Role of funding source

The funders played no role in the design, conduct or analysis of the study, nor in the interpretation or reporting of study findings, or the decision to submit it for publication.

## Results

### Sample characteristics

There were 154,490 adults aged 18+ who responded to the survey between March 2014 and August 2021 in England and 144,005 with complete cases for socio-demographic variables (no missing data for sex or age, 1·1% missing for social grade), smoking status (0·2% missing) and AUDIT score (1·3% missing).

The total sample had a weighted n of 144,583. The sample had a weighted mean age of 47·8 years (SD=18·59), 51·0% (unweighted n=72,374) were female, and 44·9% (unweighted n=60,069) were of social grade C2DE. Of the total sample, 19·1% were past year-smokers (unweighted n=26,801) and 17·6% were current smokers (unweighted n=24,811). Of the total sample, 0·6% (unweighted n=852) were at risk of alcohol dependence, 70·7% (unweighted n=100,494) were drinkers not at risk of alcohol dependence and 28·6% (unweighted n=42,659) were non-drinkers.

### Past-year and current smoking prevalence rates

**Figure 1:**
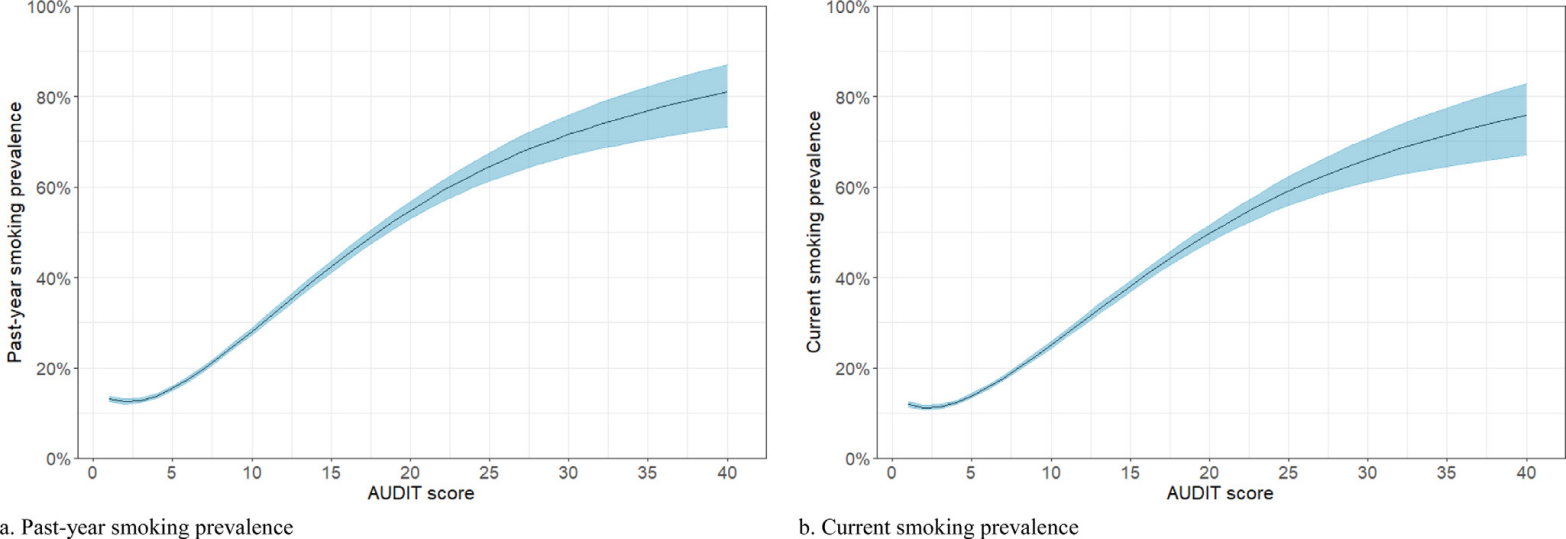
Smoking prevalence by AUDIT score among drinkers (adjusted for survey year, holding it constant at 2020 for purposes of prediction). a. Past-year smoking prevalence. b. Current smoking prevalence.

Past-year smoking prevalence was 63·3% (95% CI=59·7-66·8) among those at risk of alcohol dependence, 18·7% (95% CI=18·4-18·9) among drinkers not at risk of alcohol dependence, and 19·2% (95% CI=18·8-19·7) among non-drinkers from 2014 to 2021. Predicted values for past-year smoking prevalence rates are modelled, adjusted for survey year, across the full range of AUDIT scores among drinkers and presented in Table 1 and Figure 1.

**Table 1:** Prevalence of past-year and current smoking among drinkers (weighted n=103,162) by AUDIT score (predicted values).

AUDIT score	Past-year smoking prevalence, % (95% CI)	Current smoking prevalence, % (95% CI)
5	15·4 (14·9, 16·0)	13·8 (13·3, 14·3)
10	28·1 (27·3, 28·9)	25·1 (24·3, 25·9)
15	42·3 (41·0, 43·6)	38·0 (36·8, 39·3)
20	54·8 (52·9, 56·7)	49·8 (47·9, 51·7)
25	64·5 (61·3, 67·6)	59·1 (55·9, 62·2)
30	71·6 (66·8, 75·9)	66·1 (61·2, 70·8)
35^a^	76·9 (70·5, 82·2)	71·5 (64·6, 77·5)
40	81·1 (73·2, 87·0)	75·9 (67·2, 82·8

a Mean prevalence rate of AUDIT scores 34 and 36 taken as no respondent existed with an AUDIT score of 35 (adjusted for survey year, holding it constant at 2020 for purposes of prediction).

Current smoking prevalence was 57·9% (95% CI=54·3-61·6) among those at risk of alcohol dependence, 17·1% (95% CI=16·8-17·4) among drinkers not at risk of alcohol dependence, and 18·1% (95% CI=17·7-18·5) among non-drinkers. Predicted values for current smoking prevalence rates are modelled, adjusted for survey year, across the full range of AUDIT scores among drinkers and presented in Table 1 and Figure 1.

### Smoking and smoking cessation attempt characteristics among past-year smokers

Past-year smokers at risk of alcohol dependence smoked an average of 14·0 cigarettes per day compared with 11·5 among non-drinkers and 10·9 among drinkers not at risk, see Table 2. Nearly a third (30·6%) of those at risk of alcohol dependence reported smoking within 5 minutes of waking up compared with 13·2% of drinkers not at risk and 17·4% of non-drinkers. Nearly two-thirds (65·4%) of past-year smokers at risk of alcohol dependence smoked roll your own cigarettes compared with less than half of drinkers not at risk (49·7%) and non-drinkers (45·1%). A similar proportion of serious attempts to quit smoking (34·1% vs. 32·1% vs. 32·7%) were reported across all three groups. Quit success rates were similar among past-year smokers at risk of alcohol dependence (14·7%) and non-drinkers (14·6%) though higher among drinkers not at risk (19·8%). A higher proportion of abrupt quit attempts were made among past-year smokers at risk of alcohol dependence (67·8%) compared with drinkers not at risk (54·6%) and non-drinkers (46·5%). Smoking and smoking cessation attempt characteristics among past-year smokers show a clear dose-response relationship where there are changes in characteristics as a function of AUDIT score and are presented in Supplementary Table 1 and Supplementary Figure 1.

**Table 2:** Smoking and smoking cessation attempt characteristics among past-year smokers (unweighted).

	N	Overall, N = 26,801^1^	Non-drinker, N = 8,019^1^	Drinker not at risk of alcohol dependence, N = 18,247^1^	At risk of alcohol dependence, N = 535^1^
Time to first cigarette	26,418				
*>60 minutes*		35·3% (34·8, 35·9); (9,338)	30·1% (29·1, 31·1); (2,388)	37·8% (37·1, 38·5); (6,783)	31·5% (27·6, 35·7); (167)
*30-60 minutes*		19·1% (18·7, 19·6); (5,057)	19·7% (18·8, 20·6); (1,559)	19·1% (18·5, 19·7); (3,428)	13·2% (10·5, 16·5); (70)
*6-30 minutes*		30·7% (30·1, 31·2); (8,102)	32·8% (31·8, 33·9); (2,602)	29·9% (29·2, 30·6); (5,369)	24·7% (21·2, 28·7); (131)
*<=5 minutes*		14·8% (14·4, 15·3); (3,921)	17·4% (16·6, 18·3); (1,380)	13·2% (12·8, 13·8); (2,379)	30·6% (26·7, 34·7); (162)
Cigarettes per day	25,267	11·1 (8·36)	11·5 (8·43)	10·9 (8·16)	14·0 (12·37)
Smokes roll your own (% yes)	24,657	48·7% (48·0, 49·3); (12,001)	45·1% (44·0, 46·3); (3,328)	49·7% (49·0, 50·5); (8,346)	65·4% (61·0, 69·5); (327)
Serious attempt to quit smoking (% yes)	25,964	32·3% (31·8, 32·9); (8,399)	32·7% (31·6, 33·7); (2,526)	32·1% (31·5, 32·8); (5,696)	34·1% (30·1, 38·4); (177)
Time since start of most recent quit attempt^2^	8,363				
*Last week*		5·7% (5·2, 6·2); (478)	6·9% (5·9, 8·0); (173)	5·2% (4·7, 5·9); (297)	4·5% (2·1, 9·1); (8)
*Between a week and a month*		11·0% (10·3, 11·7); (918)	12·0% (10·7, 13·3); (301)	10·5% (9·8, 11·4); (598)	10·8% (6·8, 16·6); (19)
*1-2 months*		11·1% (10·4, 11·8); (928)	11·7% (10·5, 13·1); (295)	10·8% (10·0, 11·7); (613)	11·4% (7·2, 17·2); (20)
*2-3 months*		12·5% (11·8, 13·2); (1,046)	12·4% (11·2, 13·8); (313)	12·5% (11·6, 13·4); (708)	14·2% (9·6, 20·4); (25)
*3-6 months*		21·6% (20·7, 22·5); (1,808)	19·7% (18·2, 21·3); (496)	22·4% (21·3, 23·5); (1,268)	25·0% (18·9, 32·2); (44)
*6-12 months*		38·1% (37·0, 39·1); (3,185)	37·3% (35·4, 39·2); (938)	38·6% (37·3, 39·8); (2,187)	34·1% (27·2, 41·7); (60)
Abrupt quit attempt made^2^ (% yes)	8,362	52·4% (51·4, 53·5); (4,384)	46·5% (44·5, 48·5); (1,168)	54·6% (53·3, 55·9); (3,096)	67·8% (60·3, 74·5); (120)
Use of evidence-based aids during most recent quit attempt^2^ (% yes)	8,399	53·9% (52·8, 54·9); (4,524)	54·4% (52·4, 56·3); (1,373)	53·8% (52·5, 55·1); (3,064)	49·2% (41·6, 56·7); (87)
Receipt of GP advice and/or support^2^ (% yes)	8,399	39·2% (38·2, 40·3); (3,294)	46·0% (44·0, 48·0); (1,162)	36·1% (34·9, 37·4); (2,059)	41·2% (34·0, 48·9); (73)
Quit success^2^ (% successful)	8,399	18·1% (17·3, 19·0); (1,521)	14·6% (13·3, 16·1) (369)	19·8% (18·7, 20·8); (1,126)	14·7% (10·0, 21·0); (26)

1 % (95% CI); n; Mean (SD).

2 only asked of those who made a serious quit attempt in past 12 months.

### Differences in smoking and smoking cessation attempt characteristics among past-year smokers between those at risk of alcohol dependence and those who drink not at risk of alcohol dependence

Among past-year smokers, those drinkers at risk of alcohol dependence were more likely than those not at risk to smoke roll your own cigarettes, and, if they had made a recent quit attempt, they were more likely to have made it abruptly, see Table 3. They also smoked an average of three cigarettes more per day than drinkers not at risk. No differences were detected between drinkers at risk of alcohol dependence versus not at risk in terms of the likelihood of making a serious quit attempt and use of evidence-based aids or receipt of GP advice and/or support. Odds of quit success were 31% lower in drinkers at risk of alcohol dependence, compared with not at risk, though the confidence interval was wide and contained no difference.

**Table 3:** Smoking and smoking cessation attempt characteristics among past-year smokers predicted by risk of alcohol dependence, adjusted for survey year (held constant at the median year, 2017) (linear and logistic regression models).

	Roll your own smoker		Cigarettes per day		Made serious quit attempt		Abrupt quit attempt		Use of evidence-based aids		Received GP advice and/or support		Quit success	
	OR (95% CI)^1^	p-value	B (95% CI)	p-value	OR (95% CI)^1^	p-value	OR (95% CI)^1^	p-value	OR (95% CI)^1^	p-value	OR (95% CI)^1^	p-value	OR (95% CI)^1^	p-value
At risk of alcohol dependence (versus drinker not at risk of alcohol dependence [ref])	1·91 (1·58, 2·30)	<0·001	3·0 (2·3, 3·8)	<0·001	1·10 (0·91, 1·32)	0·318	1·76 (1·28, 2·43)	<0·001	0·83 (0·62, 1·13)	0·235	1·26 (0·92, 1·70)	0·140	0·69 (0·45, 1·04)	0·090

A linear regression models was used for cigarettes per day. Logistic regression models were used for roll your own smoker, made serious quit attempt, abrupt quit attempt, use of evidence-based aids, received GP advice and/or support, and quit success.

1 OR = Odds Ratio, CI = Confidence Interval.

Among past-year smokers, drinkers at risk of alcohol dependence were more likely than those not at risk to smoke their first cigarette within 5 minutes of waking, see Table 4. No differences were detected between drinkers at versus not at risk of alcohol dependence in the start of the most recent quit attempt being made more than a week ago versus last week.

**Table 4:** Smoking and smoking cessation attempt characteristics among past-year smokers predicted by risk of alcohol dependence, adjusted for survey year (held constant at the median year, 2017).

	Time to first cigarette (>60 minutes [ref])						Time since start of most recent quit attempt (last week [ref])									
	30-60 minutes		6-30 minutes		<=5 minutes		Between a week and a month		1-2 months		2-3 months		3-6 months		6-12 months	
	RRR (95% CI)^1^	p-value	RRR (95% CI)^1^	p-value	RRR (95% CI)^1^	p-value	RRR (95% CI)^1^	p-value	RRR (95% CI)^1^	p-value	RRR (95% CI)^1^	p-value	RRR (95% CI)^1^	p-value	RRR (95% CI)^1^	p-value
At risk of alcohol dependence (versus drinker not at risk of alcohol dependence [ref])	0·84 (0·63, 1·11)	0·216	1·01 (0·80, 1·27)	0·955	2·81 (2·25, 3·51)	<0·001	1·19 (0·51, 2·74)	0·689	1·21 (0·53, 2·79)	0·647	1·32 (0·59, 2·96)	0·501	1·29 (0·60, 2·77)	0·515	1·02 (0·48, 2·15)	0·963

Multinomial regression models were used for time to first cigarette and time since start of most recent quit attempt.

1 RRR = Relative Risk Ratio, CI = Confidence Interval.

### Sensitivity analyses among current smokers

A very similar pattern of results was found across sensitivity analyses using a sample of current smokers rather than past-year smokers (Supplementary Figure 2 and Supplementary Tables 2-5). Motivation to quit smoking was lower in drinkers at risk of alcohol dependence versus not at risk, see Supplementary Table 4.

## Discussion

### Summary of findings

Our data, collected between 2014 and 2021 from a representative survey in England, showed a strong positive relationship between alcohol consumption and smoking. Past-year smoking prevalence was 63% (current smoking prevalence of 58%) among people at risk of alcohol dependence and was 19% (current smoking, 18%) among non-drinkers. Prevalence increased across the full range of AUDIT scores, rising gradually up to an estimated 81% (current smoking, 76%) in the heaviest drinkers in 2020. While the confidence intervals were reasonably wide for both past-year and current smoking prevalence for the group of people at risk of alcohol dependence, these were still substantially higher (even at the lower boundary) than the much more precise estimate for both drinkers not at risk of alcohol dependence and non-drinkers, giving us confidence in these results.

The way drinkers smoked differed by risk of alcohol dependence in several ways. Firstly, past-year smokers who were at risk of alcohol dependence had higher levels of cigarette dependence as indicated by cigarettes smoked per day (a mean of 14, three more than drinkers not at risk) and time to first cigarette (more likely than those not at risk to report smoking within 5 minutes of waking up). Secondly, roll your own cigarettes were especially common among those at risk of alcohol dependence, with nearly two-thirds of past-year smokers reporting using them.

A third of past-year smokers at risk of alcohol dependence reported making a serious attempt to quit smoking in the past year, and of those 15% were successful. No significant differences were detected between those drinkers at risk of alcohol dependence (compared with those not at risk) in terms of the likelihood of making a serious quit attempt or success in that quit attempt. About two-thirds of quit attempts made among past-year smokers at risk of alcohol dependence were abrupt, and those drinkers at risk of alcohol dependence were more likely to have made an abrupt attempt compared with drinkers not at risk. No significant differences were detected between those drinkers at risk of alcohol dependence, compared with those not at risk, in terms of the likelihood of using evidence-based aids, receipt of GP advice and/or support, or the time since the start of the most recent quit attempt.

### Implications for practice and policy

This study has important implications for policy given that our findings show a high prevalence of past-year and current smoking among those at risk of alcohol dependence. To reach the UK Government's target of lowering smoking prevalence below 5% by 2030, efforts need to be focused on those groups where smoking is most prevalent. Our data show this should include targeted support for those at risk of alcohol dependence and that policy and practice documents need to explicitly highlight this group, alongside other intersections of disadvantage including living in social housing, mental illness, other substance dependencies and marginalised and minority communities, to improve treatment as part of the NHS long-term plan and to reduce health inequalities associated with smoking.^1^^,^^2^

There is some guidance and advice from health agencies, such as Public Health England, that support and treatment should be offered to all smokers, including those from priority groups with high smoking prevalence rates, though this is not always the case.^27^ There needs to be clear guidance around how to help those at risk of alcohol dependence who appear to be at increased risk of being excluded from smoking cessation support and may have complex and/or different treatment needs. This should include what services are best placed to provide support to those smokers at risk of alcohol dependence, which strategies work best, and how best to provide support in the community for people who may not be coming into substance use treatment. As with other areas of health (e.g. Housing First, substance harm reduction), the support offered potentially needs to be embedded within existing interventions or pathways of change, so to appreciate the wider determinants of health. For example, offering support for smoking cessation which both addresses the persons high levels of tobacco dependence (e.g. behavioural support and combination products, or e-cigarettes) while appreciating challenges in access to treatment and ability to reduce smoking within the persons lived circumstances due to heavier drinking (e.g. harm reduction versus abstinence orientated goals).

### Strengths and limitations

A key strength of this study is that it was the first to assess smoking prevalence among those at risk of alcohol dependence in the general population in England using a nationally representative survey.

A limitation is that the proportion of the total sample in this study at risk of alcohol dependence (0·6%) may have been an underestimate (the 2014 Adult Psychiatric Morbidity Survey estimated 1·4% of the adult population in England^10^). Population survey research is limited by biases introduced through the exclusion of certain populations from both the sampling frame and through non-response bias. Due to the random location sampling used in these surveys, it is not appropriate to record response rates as the interviewers have a choice as to the properties approached, and therefore it is not possible to measure this non-response bias. The populations who are usually excluded, such as people who are homeless, tend to consume on average more alcohol than the general population and have a higher smoking prevalence.^28^ These biases can lead to the underestimation of alcohol consumption at the population level.^29^ This suggests that our study may have underestimated the prevalence of being at risk of alcohol dependence in the general population in England, and furthermore, smoking prevalence in this population. Furthermore, we relied on self-reported measures of smoking and alcohol consumption and did not account for typical under-reporting, which may have differed based on how heavily people drink alcohol.

Another potential limitation is that the Covid-19 pandemic occurred during the study period. However, research has shown that the first Covid-19 lockdown was not associated with a significant change in smoking prevalence;^30^ it is therefore unlikely to have unduly affected our study results.

## Conclusions

In a representative sample of adults in England between 2014 and 2021, past-year smoking prevalence was 63% and current smoking prevalence was 58% among people who were at risk of alcohol dependence, with a graded effect where smoking prevalence increased with level of alcohol consumption. Past-year smokers who were at risk of alcohol dependence had higher levels of cigarette dependence (as indicated by cigarettes smoked per day and time to first cigarette) than those drinkers not at risk. The high smoking prevalence among those at risk of alcohol dependence has important implications for policy in that to reduce smoking prevalence in England in line with the UK Government's target of less than 5% by 2030, efforts need to be focused on this group.

## Contributors

SC led the conceptualisation of the study with all other authors supporting this. CG wrote the original draft. CG, MO, LS, HTB and SC reviewed and edited the protocol and the final manuscript. CG led the formal analysis and HTB validated the analysis and findings. CG and HTB have directly accessed and verified the underlying data reported in the manuscript. All authors (CG, MO, LS, HTB and SC) confirm that they had full access to all the data in the study and accept responsibility for the decision to submit for publication.

## Data availability statement

The deidentified participant data (with age banded to ensure anonymity of dataset), along with the study protocol including the analysis plan, are available on Open Science Framework, at https://osf.io/mbqyr/.

## Declaration of interests

CG and MO are paid scientific consultants for the behaviour change and lifestyle organisation ‘One Year No Beer’. SC and HTB have no conflicts to declare. LS is a HEFCE funded member of staff at University College London. He has received honoraria for talks, an unrestricted research grant and travel expenses to attend meetings and workshops from Pfizer and an honorarium to sit on advisory panel from Johnson&Johnson, both pharmaceutical companies that make smoking cessation products. He has acted as paid reviewer for grant awarding bodies and as a paid consultant for health care companies. Other research has been funded by the government, a community-interested company (National Centre for Smoking Cessation) and charitable sources. He has never received personal fees or research funding of any kind from alcohol, electronic cigarette or tobacco companies.

## Appendix Supplementary materials

Image, application 1

Image, application 2
