# Supplementary material for: Characterising smoking and smoking cessation attempts by risk of alcohol dependence: A representative, cross-sectional study of adults in England between 2014-2021
Source: Lancet Reg Health Eur. 2022 Jun 9;18:100418. doi: 10.1016/j.lanepe.2022.100418 (PMC9257647; doi:10.1016/j.lanepe.2022.100418)
Supplement: Supplementary file 1 [file mmc1.docx]

# Supplementary Tables

Supplementary Table 1: Smoking and smoking cessation characteristics in past-year smokers modelled for drinkers, adjusted for survey year (held constant at the median year, 2017) (n=18,771) (predicted values)

| % (95% CI^1^), unless otherwise stated | N | AUDIT score | | | | | | | |
| --- | --- | --- | --- | --- | --- | --- | --- | --- | --- |
|  |  | 5 | 10 | 15 | 20 | 25 | 30 | 35 | 40 |
| Time to first cigarette, most likely category | 18,472 | >60 minutes | >60 minutes | >60 minutes | >60 minutes | >60 minutes | <=5 minutes | <=5 minutes | <=5 minutes |
| Cigarettes per day, mean (95% CI) | 17,655 | 10·9 (10·6, 11·1) | 10·7 (10·5, 11·0) | 11·1 (10·8, 11·5) | 12·2 (11·8, 12·6) | 13·8 (13·2, 14·5) | 15·9 (14·9, 16·9) | 18·2 (16·6, 19·8) | 20·7 (18·5, 22·8) |
| Smokes roll your own | 17,266 | 50·1 (48·7, 51·6) | 55·5 (54·0, 57·0) | 60·1 (58·0, 62·1) | 64·1 (61·7, 66·5) | 67·7 (64·1, 71·2) | 71·0 (65·1, 76·3) | 73·9 (65·1, 81·1) | 76·6 (64·7, 85·4) |
| Serious attempt to quit smoking | 18,218 | 30·2 (28·9, 31·6) | 30·3 (29·0, 31·7) | 30·7 (28·8, 32·6) | 30·6 (28·4, 32·9) | 30·1 (26·9, 33·6) | 29·4 (24·2, 35·2) | 28·5 (21·0, 37·4) | 27·5 (17·8, 40·0) |
| Time since start of most recent quit attempt, most likely category | 5,842 | 6-12 months | 6-12 months | 6-12 months | 6-12 months | 6-12 months | 6-12 months | 6-12 months | 6-12 months |
| Abrupt quit attempt made | 5,844 | 54·8 (52·3, 57·4) | 57·0 (54·4, 59·6) | 59·7 (56·1, 63·2) | 63·0 (58·7, 67·2) | 66·8 (60·0, 73·1) | 70·8 (59·4, 80·1) | 74·6 (57·9, 86·2) | 78·1 (56·1, 90·8) |
| Use of evidence-based aids during most recent quit attempt | 5,867 | 55·0 (52·4, 57·5) | 54·8 (52·1, 57·4) | 53·3 (49·6, 56·9) | 52·5 (48·2, 56·8) | 52·4 (45·5, 59·2) | 52·6 (41·0, 63·9) | 53·0 (35·8, 69·5) | 53·5 (30·7, 74·8) |
| Receipt of GP advice and/or support | 5,867 | 39·0 (36·5, 41·6) | 35·6 (33·1, 38·2) | 36·0 (32·6, 39·6) | 39·5 (35·3, 43·9) | 45·4 (38·6, 52·5) | 53·1 (41·3, 64·6) | 61·4 (43·8, 76·5) | 69·2 (46·0, 85·6) |
| Quit success | 5,867 | 19·0 (17·1, 21·1) | 18·4 (16·4, 20·5) | 16·4 (14·0, 19·1) | 14·4 (11·7, 17·6) | 12·6 (8·7, 17·8) | 10·9 (5·7, 19·6) | 9·3 (3·5, 22·5) | 8·0 (2·1, 25·9) |

^1^ CI: Confidence Interval

Supplementary Table 2: Smoking and smoking cessation characteristics in current smokers (unweighted)

| % (n), unless otherwise stated | N | Overall, N = 24,811 | Non-drinker, N = 7,554 | Drinker not at risk of alcohol dependence, N = 16,763 | At risk of alcohol dependence, N = 494 |
| --- | --- | --- | --- | --- | --- |
| Time to first cigarette | 24,483 |  |  |  |  |
| *>60 minutes* |  | 34·8% (34·2, 35·4); (8,520) | 29·8% (28·8, 30·9); (2,233) | 37·2% (36·5, 38·0); (6,144) | 29·1% (25·2, 33·4); (143) |
| *30-60 minutes* |  | 19·3% (18·8, 19·8); (4,722) | 19·7% (18·9, 20·7); (1,477) | 19·3% (18·7, 19·9); (3,179) | 13·4% (10·6, 16·9); (66) |
| *6-30 minutes* |  | 31·1% (30·5, 31·6); (7,604) | 33·1% (32·0, 34·2); (2,476) | 30·3% (29·6, 31·0); (5,001) | 25·9% (22·1, 30·0); (127) |
| *<=5 minutes* |  | 14·9% (14·4, 15·3); (3,637) | 17·3% (16·5, 18·2); (1,295) | 13·2% (12·7, 13·8); (2,187) | 31·6% (27·5, 35·9); (155) |
| Cigarettes per day, mean (SD) | 23,420 | 11·0 (8·17) | 11·3 (8·10) | 10·8 (8·02) | 14·3 (12·53) |
| Smokes roll your own | 22,868 | 49·3% (48·7, 50·0); (11,274) | 45·5% (44·3, 46·7); (3,170) | 50·5% (49·7, 51·3); (7,791) | 67·6% (63·1, 71·8); (313) |
| Serious attempt to quit smoking | 24,023 | 28·1% (27·5, 28·6); (6,741) | 29·5% (28·5, 30·6); (2,150) | 27·3% (26·7, 28·0); (4,448) | 29·9% (25·8, 34·2); (143) |
| Time since start of most recent quit attempt | 6,710 |  |  |  |  |
| *Last week* |  | 6·2% (5·7, 6·8); (418) | 7·5% (6·4, 8·7); (160) | 5·7% (5·0, 6·4); (251) | 4·9% (2·2, 10·2); (7) |
| *Between a week and a month* |  | 11·3% (10·5, 12·1); (757) | 12·4% (11·0, 13·9); (265) | 10·7% (9·8, 11·7); (474) | 12·6% (7·8, 19·4); (18) |
| *1-2 months* |  | 11·8% (11·1, 12·7); (795) | 12·4% (11·0, 13·9); (265) | 11·6% (10·7, 12·6); (514) | 11·2% (6·7, 17·8); (16) |
| *2-3 months* |  | 13·1% (12·3, 14·0); (881) | 13·3% (11·9, 14·8); (285) | 12·9% (12·0, 14·0); (573) | 16·1% (10·7, 23·4); (23) |
| *3-6 months* |  | 21·3% (20·3, 22·3); (1,426) | 19·1% (17·5, 20·9); (409) | 22·2% (21·0, 23·5); (984) | 23·1% (16·6, 31·0); (33) |
| *6-12 months* |  | 36·3% (35·1, 37·4); (2,433) | 35·3% (33·3, 37·4); (756) | 36·8% (35·4, 38·3); (1,631) | 32·2% (24·7, 40·6); (46) |
| Abrupt quit attempt made | 6,704 | 47·6% (46·4, 48·8); (3,188) | 41·7% (39·6, 43·9); (892) | 49·8% (48·3, 51·3); (2,204) | 64·3% (55·8, 72·0); (92) |
| Use of evidence-based aids during most recent quit attempt | 6,741 | 54·0% (52·8, 55·2); (3,641) | 54·7% (52·6, 56·8); (1,176) | 53·9% (52·4, 55·3); (2,396) | 48·3% (39·9, 56·7); (69) |
| Receipt of GP advice and/or support | 6,741 | 41·8% (40·6; 43·0); (2,816) | 48·0% (45·8, 50·1); (1,031) | 38·6% (37·2, 40·1); (1,719) | 46·2% (37·9, 54·7); (66) |
| Motivation to quit smoking^1^, mean (SD) | 24,654 | 3·2 (1·97) | 3·2 (2·05) | 3·2 (1·94) | 2·9 (1·87) |
| ^1^ 1=Do not want to stop smoking; 7=Really want to stop smoking and intend to in the next month | | | | | |

Supplementary Table 3: Smoking and smoking cessation characteristics in current smokers modelled for drinkers, adjusted for survey year (held constant at the median year, 2017) (n=17,246) (predicted values)

|  | N | AUDIT score | | | | | | | |
| --- | --- | --- | --- | --- | --- | --- | --- | --- | --- |
|  |  | 5 | 10 | 15 | 20 | 25 | 30 | 35 | 40 |
| Time to first cigarette | 16,987 | >60 minutes | >60 minutes | >60 minutes | >60 minutes | <=5 minutes | <=5 minutes | <=5 minutes | <=5 minutes |
| Cigarettes per day, mean (95% CI) | 16,234 | 10·7 (10·5, 11·0) | 10·6 (10·4, 10·9) | 11·1 (10·8, 11·5) | 12·3 (11·9, 12·7) | 14·0 (13·4, 14·7) | 16·2 (15·2, 17·2) | 18·6 (17·1, 20·2) | 21·2 (19·0, 23·4) |
| Smokes roll your own, % (95% CI) | 15,887 | 50·6 (49·0, 52·1) | 55·7 (54·1, 57·2) | 60·5 (58·4, 62·6) | 65·0 (62·5, 67·5) | 69·2 (65·4, 72·7) | 73·0 (67·0, 78·2) | 76·4 (67·7, 83·4) | 79·6 (68·0, 87·7) |
| Serious attempt to quit smoking, % (95% CI) | 16,731 | 26·1 (24·8, 27·4) | 26·2 (24·9, 27·5) | 27·0 (25·2, 28·9) | 27·4 (25·1, 29·7) | 27·3 (24·1, 30·8) | 27·0 (21·8, 32·8) | 26·5 (19·0, 35·6) | 25·9 (16·1, 38·7) |
| Time since start of most recent quit attempt | 4,566 | 6-12 months | 6-12 months | 6-12 months | 6-12 months | 6-12 months | 6-12 months | 6-12 months | 6-12 months |
| Abrupt quit attempt made, % (95% CI) | 4,563 | 49·4 (46·5, 52·3) | 53·4 (50·4, 56·3) | 57·1 (53·0, 61·1) | 60·6 (55·8, 65·2) | 64·0 (56·5, 70·9) | 67·2 (54·7, 77·6) | 70·2 (51·6, 83·9) | 73·0 (48·1, 88·8) |
| Use of evidence-based aids during most recent quit attempt, % (95% CI) | 4,586 | 55·0 (52·1, 57·8) | 53·4 (50·5, 56·4) | 50·9 (46·9, 55·0) | 50·3 (45·5, 55·1) | 51·1 (43·7, 58·5) | 52·9 (40·5, 65·0) | 55·1 (36·6, 72·3) | 57·4 (32·7, 78·9) |
| Receipt of GP advice and/or support, % (95% CI) | 4,586 | 42·2 (39·4, 45·2) | 37·4 (34·6, 40·3) | 36·9 (33·0, 40·9) | 40·6 (35·9, 45·5) | 47·7 (40·2, 55·4) | 57·2 (44·5, 69·0) | 67·2 (48·6, 81·6) | 75·9 (52·6, 90·0) |
| Motivation to quit smoking^a^, mean (95% CI) | 17,124 | 3·1 (3·1, 3·2) | 3·1 (3·1, 3·2) | 3·1 (3·0, 3·2) | 3·1 (3·0, 3·2) | 2·9 (2·8, 3·1) | 2·8 (2·6, 3·0) | 2·6 (2·3, 3·0) | 2·4 (1·9, 2·9) |

^a^ Continuous score from 1 to 7, where 1=Do not want to stop smoking; 7=Really want to stop smoking and intend to in the next month

Supplementary Table 4: Smoking and smoking cessation characteristics in current smokers predicted by at risk of alcohol dependence, adjusted for survey year (held constant at the median year, 2017) (linear and logistic regression models)

|  | Roll your own smoker | | Cigarettes per day | | Made serious quit attempt | | Abrupt quit attempt | | Use of evidence-based aids | | Received GP advice and/or support | | Motivation to quit smoking | |
| --- | --- | --- | --- | --- | --- | --- | --- | --- | --- | --- | --- | --- | --- | --- |
|  | OR (95% CI)^1^ | p-value | B (95% CI) | p-value | OR (95% CI)^1^ | p-value | OR (95% CI)^1^ | p-value | OR (95% CI)^1^ | p-value | OR (95% CI)^1^ | p-value | B (95% CI) | p-value |
| At risk of alcohol dependence (versus drinker not at risk of alcohol dependence) | 2·05 (1·68, 2·50) | <0·001 | 3·5 (2·8, 4·3) | <0·001 | 1·14 (0·93, 1·39) | 0·200 | 1·82 (1·29, 2·59) | <0·001 | 0·80 (0·57, 1·11) | 0·180 | 1·37 (0·97, 1·91) | 0·070 | -0·23 (-0·40,  -0·05) | 0·010 |
| ^1^OR = Odds Ratio, CI = Confidence Interval | | | | | | | | | | | | | | |

Supplementary Table 5: Smoking and smoking cessation characteristics in current smokers predicted by at risk of alcohol dependence, adjusted for survey year (held constant at the median year, 2017) (multinomial regression models)

|  | Time to first cigarette (>60 minutes [ref]) | | | | | | Time since start of most recent quit attempt (last week [ref]) | | | | | | | | | |
| --- | --- | --- | --- | --- | --- | --- | --- | --- | --- | --- | --- | --- | --- | --- | --- | --- |
|  | 30-60 minutes | | 6-30 minutes | | <=5 minutes | | Between a week and a month | | 1-2 months | | 2-3 months | | 3-6 months | | 6-12 months | |
|  | RRR (95% CI)^1^ | p-value | RRR (95% CI)^1^ | p-value | RRR (95% CI)^1^ | p-value | RRR (95% CI)^1^ | p-value | RRR (95% CI)^1^ | p-value | RRR (95% CI)^1^ | p-value | RRR (95% CI)^1^ | p-value | RRR (95% CI)^1^ | p-value |
| At risk of alcohol dependence (versus drinker not at risk of alcohol dependence [ref]) | 0·90 (0·67, 1·21) | 0·491 | 1·11 (0·87, 1·41) | 0·405 | 3·09 (2·44, 3·90) | <0·001 | 1·37 (0·56, 3·32) | 0·490 | 1·12 (0·45, 2·75) | 0·808 | 1·45 (0·61, 3·42) | 0·400 | 1·21 (0·53, 2·76) | 0·657 | 1·01 (0·45, 2·27) | 0·974 |
| ^1^OR = Odds Ratio, CI = Confidence Interval | | | | | | | | | | | | | | |  |  |
