# Supplementary material for: Characterising smoking and smoking cessation attempts by risk of alcohol dependence: A representative, cross-sectional study of adults in England between 2014-2021
Source: Lancet Reg Health Eur. 2022 Jun 9;18:100418. doi: 10.1016/j.lanepe.2022.100418 (PMC9257647; doi:10.1016/j.lanepe.2022.100418)
Supplement: Supplementary file 2 [file mmc2.docx]

**Supplementary Figures**

| 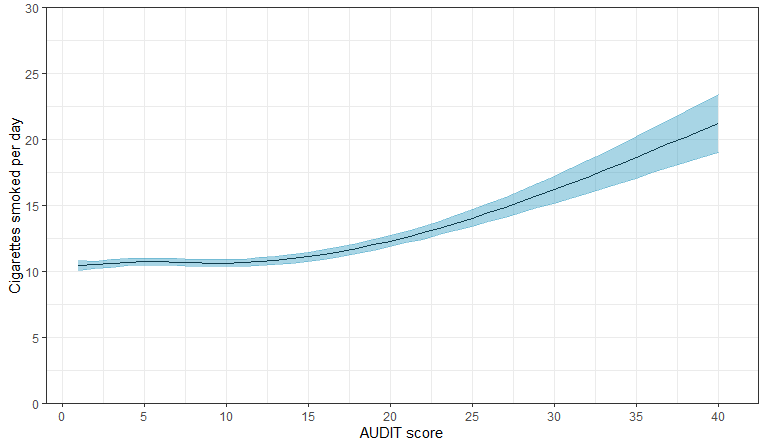 | 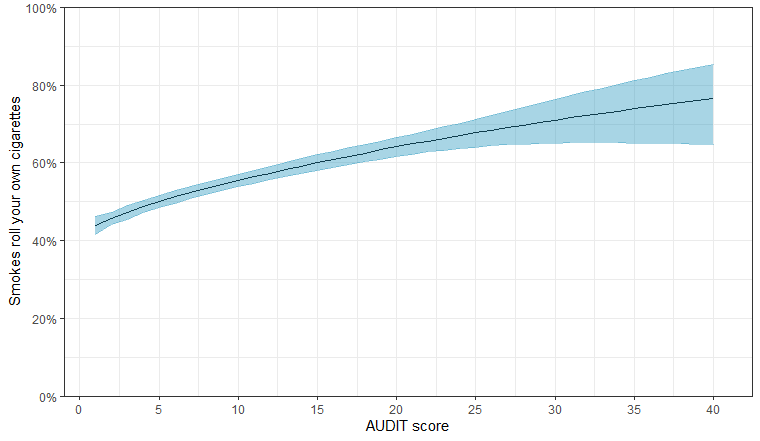 |
| --- | --- |
| a) Cigarettes smoked per day | b) Smokes roll your own cigarettes |
| 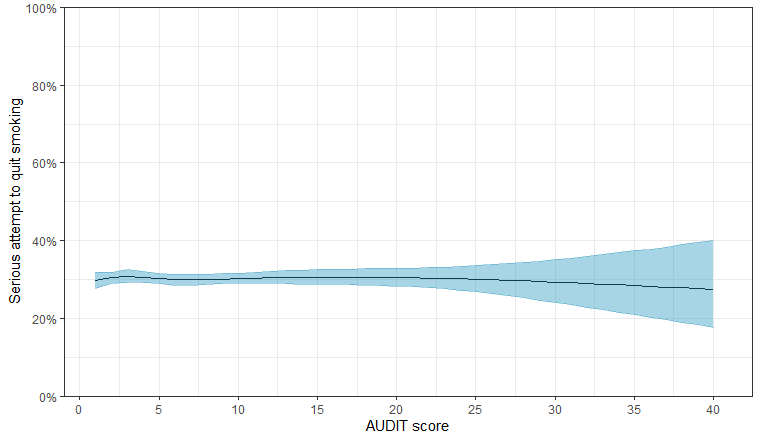 | 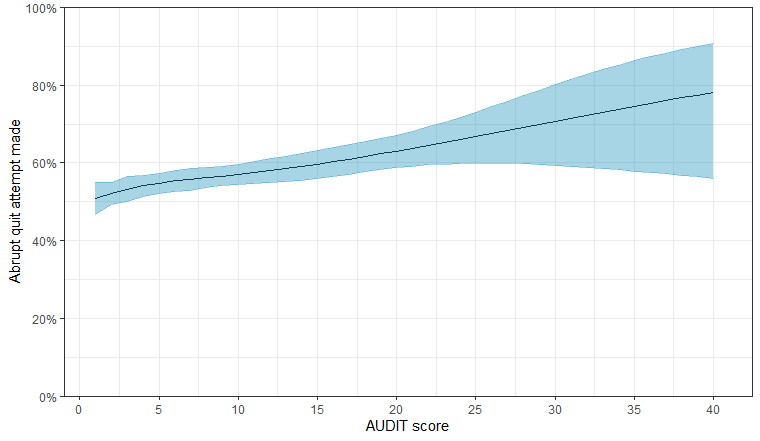 |
| c) Made a serious attempt to quit smoking | d) Made an abrupt quit attempt |
| 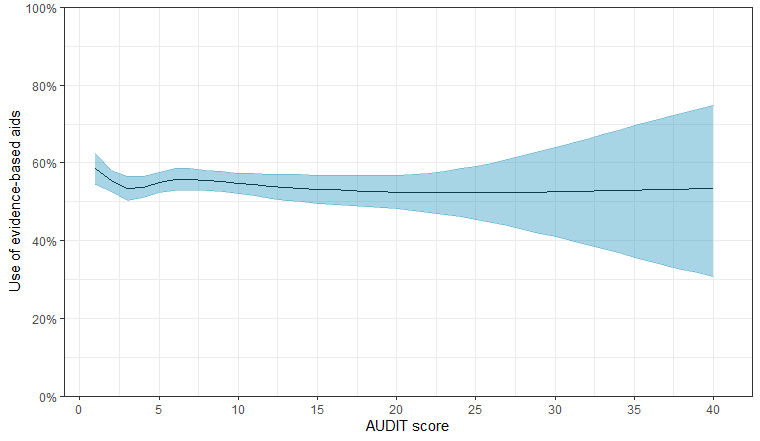 | 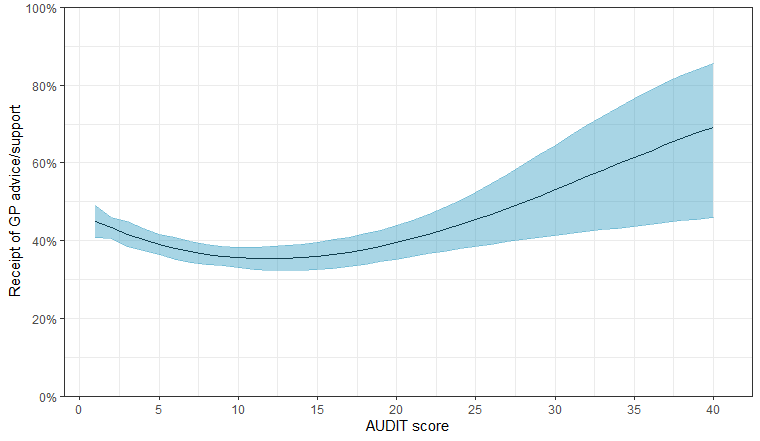 |
| e) Use of evidence-based aids during quit attempt | f) Receipt of GP advice and/or support |
| 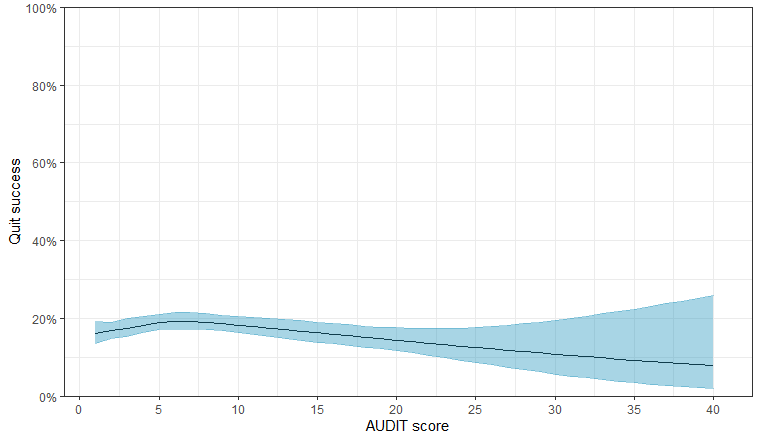 |  |
| g) Quit success (of those making a quit attempt) |  |

Supplementary Figure 1: Panel of figures for smoking and smoking cessation characteristics in past-year smokers modelled for drinkers, adjusted for survey year (held constant at the median year, 2017)

| 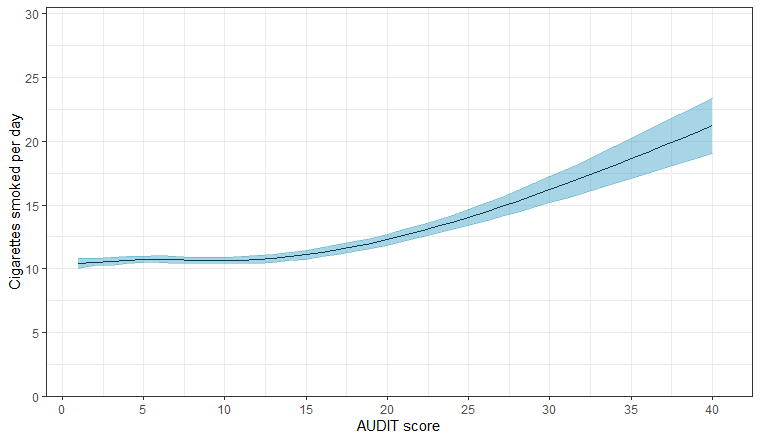 | 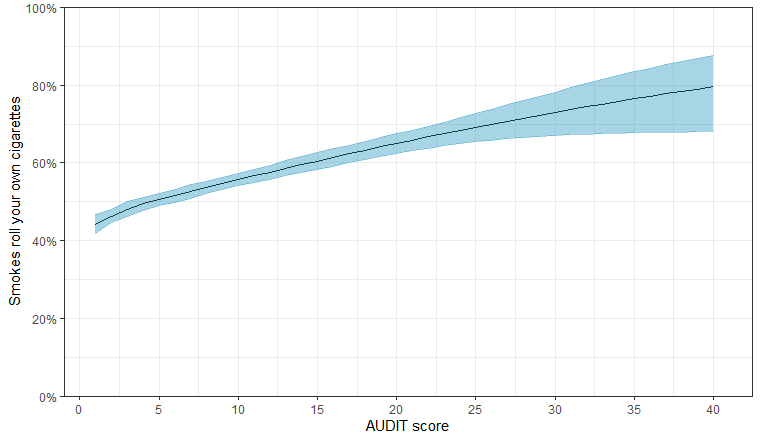 |
| --- | --- |
| a) Cigarettes smoked per day | b) Smokes roll your own cigarettes |
| 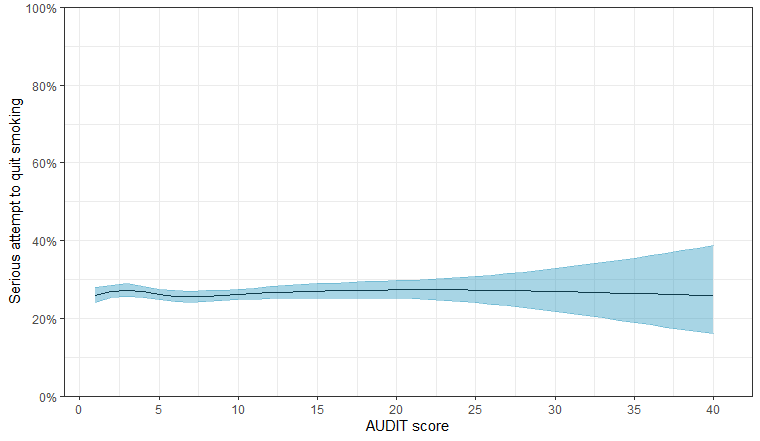 | 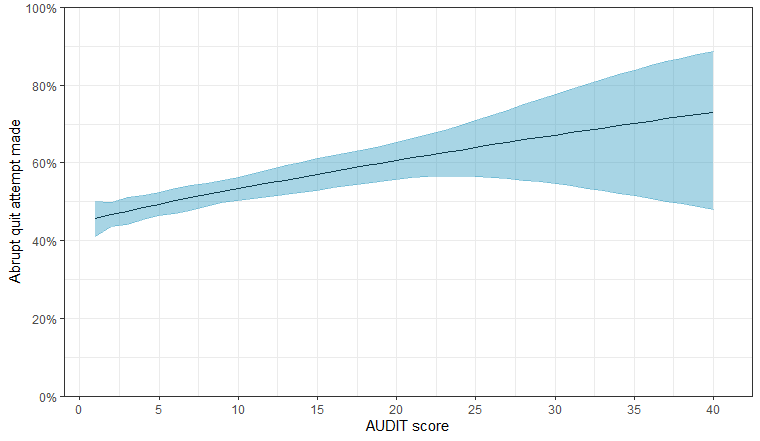 |
| c) Made a serious attempt to quit smoking | d) Made an abrupt quit attempt |
| 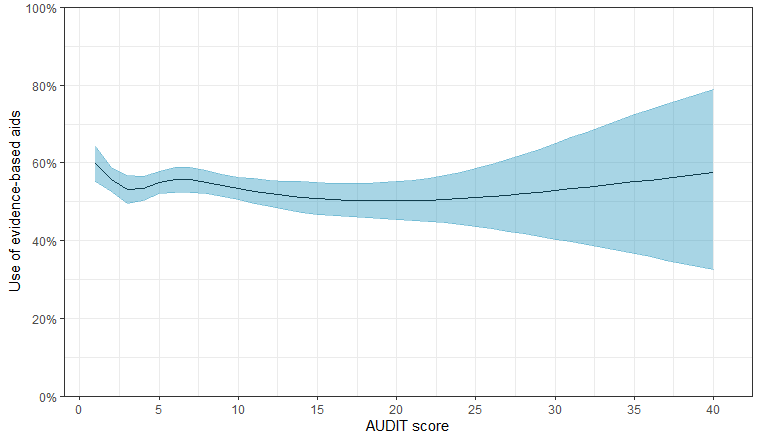 | 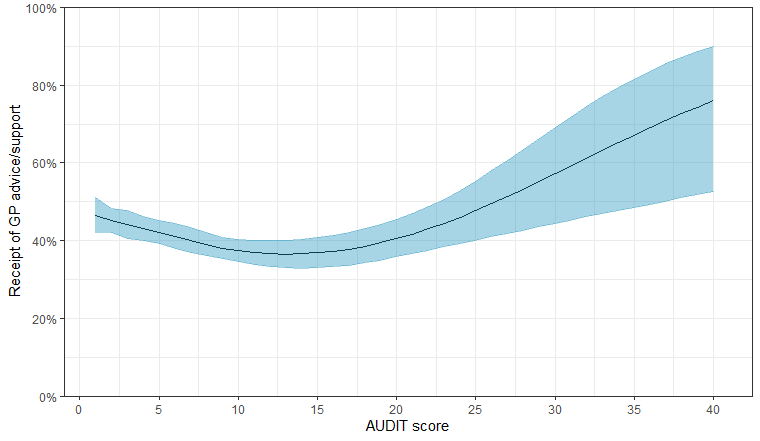 |
| e) Use of evidence-based aids during quit attempt | f) Receipt of GP advice and/or support |
| 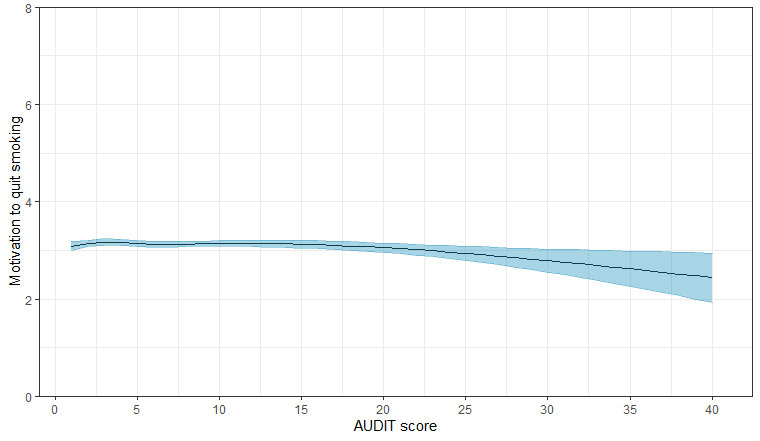 |  |
| g) Motivation to quit smoking  (continuous score from 1 to 7, where 1=Do not want to stop smoking; 7=Really want to stop smoking and intend to in the next month) | |

Supplementary Figure 2: Panel of figures for smoking and smoking cessation characteristics in current smokers modelled for drinkers, adjusted for survey year (held constant at the median year, 2017)
